# Family function and adolescent altruistic behavior: the chain mediating role of extraversion personality and perceived social support

**DOI:** 10.3389/fpsyg.2024.1431978

**Published:** 2024-10-09

**Authors:** Tao Gao, Hongbo Cui, Xiaoyan Bi, Zaihua Qing, Weiyu Chen, Keke Shi, Meidan Song, Yankun Ma

**Affiliations:** ^1^School of Education, Guangzhou University, Guangzhou, Guangdong, China; ^2^Guangdong Industry Polytechnic University, Guangzhou, China; ^3^Hunan University of Finance and Economics, Changsha, Hunan, China; ^4^Foshan Huaqiao Middle School, Foshan, China

**Keywords:** family function, extraversion personality, perceived social support, altruistic behavior, mediating effects

## Abstract

**Introduction:**

This study aimed to explore the relationship between family function and altruistic behavior in adolescents, while also investigating the potential mediating roles of extraverted personality and perceived social support.

**Methods:**

Surveys were administered to high school students across various schools in Guangdong Province, utilizing the Family APGAR Questionnaire, Extraversion Personality Scale, Perceived Social Support Scale, and Altruistic Behavior Scale. A total of 972 valid questionnaires were collected for analysis.

**Results:**

The findings revealed that family function did not directly predict altruistic behavior. However, extraverted personality and perceived social support were identified as mediators between family function and altruistic behavior. Furthermore, both extraverted personality and perceived social support acted as chain mediators in this relationship.

**Discussion:**

These results emphasize the importance of considering family dynamics in understanding adolescent altruistic behavior and highlight the potential pathways through which family function influences altruistic tendencies. Promoting positive family environments and enhancing extraverted personality and perceived social support may facilitate the cultivation of altruistic behavior in adolescents.

## Introduction

The concept of the positive adolescent development, which emerged in the 1990s, has significantly reshaped theories and practices within psychology, education, social services, and public health. This shift represents a move away from outdated problem-centered models toward a more comprehensive understanding of adolescent growth. Traditional defect model, which often paint adolescents in a negative light, can perpetuate stereotypes and exacerbate biases (Guo et al., 2017). In contrast, contemporary approaches emphasize the importance of cultivating positive traits such as altruism during this critical developmental phase.

Altruistic behavior, characterized by selfless acts of kindness devoid of any expectation of reciprocation (Kaiser, 2017; Li et al., 2020), embodies a noble trait within individuals. It epitomizes selflessness and contributes to the fostering of a morally upright society (Shi, 2020; Feng et al., 2022; Li and Wang, 2020; Song and Cao, 2022; Trivers, 1971). Understanding the factors that influence altruistic behavior among adolescents is essential for promoting positive youth development and nurturing prosocial conduct. This study delves into the influence of family functioning, extraversion personality, and perceived social support on adolescent altruism, drawing from both theoretical frameworks and empirical evidence.

### Family function and adolescent altruistic behavior

Some studies have explored how family functioning influences adolescent altruistic behavior, focusing on the role of emotional climate, social learning, and broader family dynamics. As the primary environment before adulthood, the family profoundly shapes an individual’s socialization and behavioral development (Markiewicz et al., 2001). The McMaster Family function model highlights the importance of healthy family dynamics in promoting individual well-being and positive behavior (Wang et al., 2021; Cui et al., 2022). The emotional climate within the family, characterized by warmth, support, and effective communication, directly impacts adolescents’ emotional well-being and social competence (Wang et al., 2021). Moreover, adolescents raised in supportive family environments characterized by warmth, cohesion, and effective communication are more likely to exhibit altruistic behavior (Cui et al., 2022; Jiang et al., 2018; Miller et al., 2015).

Family function affects adolescent development through several mechanisms. Social learning theory posits that parents act as primary role models, shaping their children’s behavior through observation, imitation, and reinforcement (Markiewicz et al., 2001). In families where altruistic behavior is valued and practiced, adolescents are more likely to adopt and practice these behaviors themselves. Moreover, the socialization process within the family context shapes adolescents’ social competence and interpersonal skills, further influencing their propensity for altruistic behavior. In families with open communication, cooperation, and effective conflict resolution, adolescents develop crucial skills such as empathy and perspective-taking (Eisenberg et al., 2006). These interpersonal skills are essential for understanding others’ needs and motivations, thereby facilitating altruistic actions in various social situations.

Research has consistently shown that sound Family function is associated with positive outcomes for adolescents, including higher levels of self-esteem, emotional resilience, and prosocial behavior (Cui et al., 2022). Conversely, dysfunctional family environments marked by conflict, neglect, or abuse can undermine adolescents’ development and increase their vulnerability to behavioral problems and mental health issues (Jiang et al., 2018). In conclusion, promoting healthy family dynamics is essential for fostering altruistic behavior and overall well-being among adolescents. Thus, based on previous research and theoretical frameworks, we hypothesized that positive family function will have a significant predictive effect on the development of altruistic behavior in adolescents. By fostering a supportive family environment, parents can directly influence their children’s propensity for prosocial actions.

### Extraversion personality as a mediator

In accordance with ecosystem theory, individual development is shaped by a multitude of environmental systems, with the family serving as one of the most direct and influential environmental systems. Consequently, the development of individual behavior is intricately linked to factors such as emotional connections and communication patterns within the family unit (Bronfenbrenner, 1986). In recent years, there has been a notable shift in research paradigm toward viewing the family as a holistic system, leading to increased attention on family functions, which have rapidly emerged as a focal point of research interest (Jiang et al., 2016).

The McMaster family function model theory asserts that the effective functioning of the family system profoundly impacts individuals’ physical and mental well-being, as well as their ability to navigate emotional and behavioral challenges. The fulfillment of basic family functions ensures that members’ needs are met across various domains, encompassing physical health, mental well-being, interpersonal relationships, and economic stability (Fang et al., 2004). A well-functioning family fosters warm emotional connections and healthy communication patterns, creating an environment that supports positive emotional experiences and overall well-being (Wang and Wang, 2020). Furthermore, such an environment facilitates the development of positive character traits and personality attributes, making it easier for individuals to cultivate an extroverted personality (Xie and Wu, 2021). Extraversion not only significantly predicts online altruistic behavior (Zheng and Gu, 2012) but also often leads to altruistic actions in everyday life (Oda et al., 2014).

The influence of family function extends beyond basic needs fulfillment to encompass the development of personality traits (Wei and Chen, 2010; Li, 2013). Personality traits play a pivotal role in shaping altruistic tendencies, with extraversion personality emerging as a key mediator in the relationship between family function and prosocial tendencies (Wei and Chen, 2010). Extraverted individuals exhibit traits such as sociability, assertiveness, and positive affect, predisposing them to engage in prosocial behaviors such as helping and volunteering (McCrae et al., 1999). Adolescents raised in families that foster social interaction and emotional expression are more likely to develop extraverted traits, which, in turn, facilitate altruistic behavior.

Extraverted individuals tend to engage in frequent and meaningful social interactions, creating more opportunities to demonstrate altruism. Their comfort in social settings allows them to form strong interpersonal relationships and better recognize the needs of others. Furthermore, extraverts often express their emotions openly and positively, making them approachable and more likely to be perceived as supportive by their peers. This emotional expressiveness fosters a deeper sense of connection with others, increasing the likelihood of engaging in prosocial behaviors like helping, sharing, and comforting. Research has shown that individuals who display positive emotions and are socially engaged tend to exhibit higher levels of empathy and concern for others, further driving their inclination to act altruistically (Costa and McCrae, 1992).

The influence of family function on personality development has been extensively researched, consistently demonstrating associations between family dynamics and personality traits in adolescents. Factors such as the quality of parent–child relationships, parental warmth, and parental monitoring have been identified as significant predictors of adolescent personality development (Belsky et al., 2007; Prinzie et al., 2004). Adolescents who perceive their parents as supportive, nurturing, and emotionally available are more likely to develop positive personality traits conducive to altruistic behavior.

### Perceived social support as a mediator

Perceived social support may play a critical role as a mediator between Family function and altruistic behavior, encompassing various forms of support from family, friends, and community networks (Li, 2013). Adolescents who perceive greater support from their families are more likely to exhibit altruistic tendencies, driven by a sense of security and belonging (Li et al., 2023). The theoretical frameworks of social norm theory and resource conservation theory offer insights into the mechanisms through which perceived social support influences altruistic behavior, highlighting the significance of social norms and the acquisition of valuable resources in fostering prosocial tendencies (Cialdini and Trost, 1998; Hobfoll, 1989).

Social norm theory postulates that individuals are guided by societal expectations, encouraging them to maintain a balance between giving and receiving within social relationships. Social norms refer to the behavioral standards and rules accepted and followed by members in a group (Cialdini et al., 1990). When individuals perceive care, respect, interpersonal resources, and emotional support from others—essentially, when they feel a higher level of social support—they feel compelled by these norms to reciprocate, helping both those who support them and those in need. This sense of obligation encourages prosocial behavior, including acts of altruism. In this context, social norms help explain the connection between family function and altruistic behavior, as supportive family environments foster social support, which in turn drives adolescents to engage in altruistic acts.

On the other hand, resource conservation theory suggests that individuals actively seek, preserve, and generate the resources they value (Hobfoll, 1989). Those with ample initial resources not only safeguard and maintain existing ones but also possess the capability to acquire additional resources from external sources through resource investment. Throughout this process, individuals experience a corresponding sense of well-being. When family functions effectively, adolescents are provided with a range of valuable resources spanning physiological, psychological, and social dimensions. Consequently, individuals gain a deeper understanding of the vital psychological resource of social support from the broader community. This acquisition of support contributes to their overall sense of well-being, with happier individuals demonstrating a greater propensity for engaging in altruistic behavior (Meyzari Ali and Dasht Bozorgi, 2016; Song and Wang, 2020; Wang and Zhang, 2012).

Adolescents draw upon perceived social support as a vital source of emotional validation, instrumental assistance, and social connectedness, all of which are fundamental to their overall well-being and adaptive functioning (Li, 2013). Within the context of altruistic behavior, perceived social support serves as a facilitator, providing individuals with the confidence, encouragement, and resources necessary to engage in acts of kindness and assistance (Li et al., 2023). Further research has shown that perceived support from one’s social network is closely linked to prosocial behavior (Esparza-Reig et al., 2022). This makes perceived social support a significant predictor of both prosocial and altruistic behavior (Esparza-Reig, 2020; Pastor et al., 2024; Simpson et al., 2018). Social support not only fosters individual well-being but also plays a crucial role in promoting prosocial actions, which are vital for the healthy functioning of society (Esparza-Reig et al., 2022). Furthermore, adolescents who perceive support from their social networks are more inclined to reciprocate and extend help to others, thus fostering altruistic tendencies (Ma et al., 2024) and perpetuating a cycle of mutual assistance and prosocial behavior (Cialdini and Trost, 1998).

In the present study, we hypothesized that extraversion personality and perceived social support serve as parallel mediators between family function and altruistic Behavior. This hypothesis is grounded in both psychological and sociological frameworks that link family dynamics, personality development, and social support networks with prosocial behaviors.

### Extraversion personality and perceived social support as chain mediation

The chain mediation model involving extraversion and perceived social support offers a deeper understanding of the mechanisms underlying adolescent altruistic behavior. Bandura’s (1978) ternary interaction determinism suggests that the interaction between environmental factors and individual subjective factors determines behavior. In this context, family function, as an environmental factor, can impact altruistic behavior indirectly through the mediating roles of extraversion and perceived social support. Specifically, family function shapes extraversion, which then influences perceived social support, and together, they drive altruistic tendencies.

Family function significantly influences the formation of extraversion personality (Wei and Chen, 2010). Extraverted individuals, characterized by sociability and positive emotional expression, are more likely to engage in social interactions, thereby expanding their perceived social support networks (McCrae et al., 1999). This enhanced social network, in turn, reinforces their altruistic behavior by providing emotional and instrumental support. Perceived social support serves as a facilitator that encourages individuals to act altruistically, as they are more inclined to reciprocate support and engage in prosocial actions (Li, 2013; Wei and Chen, 2010). Thus, extraversion and perceived social support form a sequential pathway—extraversion leads to greater perceived social support, which, in turn, fosters altruistic behavior.

The synergistic effects of extraversion personality and perceived social support underscore the importance of considering both individual and contextual factors in understanding altruistic behavior. Adolescents who possess extraverted traits and perceive support from their social networks are more likely to engage in altruistic acts, driven by their inherent sociability, positive affect, and sense of belonging (McCrae et al., 1999; Li, 2013). Moreover, the reciprocal relationship between extraversion personality and perceived social support amplifies the positive outcomes associated with prosocial behavior, including enhanced well-being, social integration, and adaptive functioning (Wei and Chen, 2010; Li, 2013). This reciprocal reinforcement may further strengthen the emergence of altruistic behavior.

In conclusion, the interplay of Family function, extraversion personality, and perceived social support influences adolescent altruistic behavior, highlighting the importance of considering both interpersonal and intrapersonal factors in promoting prosocial behavior. By fostering supportive family environments, cultivating positive personality traits, and strengthening social support networks, policymakers, educators, and practitioners can contribute to promoting altruism and the well-being of adolescents in society. In the present study, we hypothesized that extraversion personality and perceived social support also serve as chain mediators between family function and altruistic behavior. This hypothesis posits that the influence of family function on altruistic behavior occurs through a sequential pathway—first by shaping extraverted personality traits and then by enhancing the perception of social support, which together foster altruistic tendencies.

**Figure 1:**
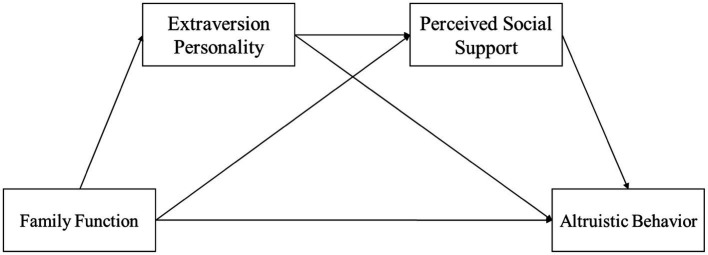
The hypothesized model.

Drawing upon the preceding discussion, this study posits the following three hypotheses: (1) Family Function has a positive predictive effect on Altruistic Behavior; (2) extraversion personality and Perceived Social Support serve as parallel mediators between Family Function and Altruistic Behavior; (3) extraversion personality and Perceived Social Support also serve as a chain mediating between Family Function and Altruistic Behavior. The conceptual model representing these hypotheses is depicted in Figure 1.

## Materials and methods

### Participants

This study aimed to explore the factors influencing adolescent altruistic behavior, focusing on the role of family function, extraversion personality, and perceived social support. High schools in Guangzhou and Foshan, two major cities in Guangdong Province, were chosen for their diverse student populations and accessibility for the study’s cluster sampling method. These locations were selected to provide a representative sample of urban adolescents in southern China.

Informed consent was obtained from school authorities and students before employing cluster sampling to select participants from one high school each in Guangzhou and Foshan, Guangdong Province. Collective testing was conducted on a class basis, resulting in 972 valid questionnaires out of 1,060 distributed, yielding an effective rate of 91.7%. The average age of the participants was 16.74 ± 1.01 years, with 400 male students (41.15%) and 572 female students (58.85%). Additionally, 252 participants were only children (25.93%), while 720 were non-only children (74.07%).

## Measures

### Family APGAR questionnaire

This questionnaire, originally developed by Smilkstein in 1978 and introduced to China by Lv and Gu (1995), was chosen for its ability to effectively assess family functioning across five key dimensions: family adaptability, cooperation, growth, emotion, and intimacy. This measure aligns with the study’s objective of understanding how family dynamics influence adolescent altruistic behavior. Respondents rate their agreement on a 3-point scale: “often like this” (2 points), “sometimes like this” (1 point), and “almost rarely” (0 points). The total score, obtained by summing the scores of the five questions, ranges from 0 to 10. Scores falling within 7–10 points indicate good family function, 4–6 points indicate moderate family function impairment, and 0–3 points indicate severe family function impairment. The questionnaire demonstrates good internal consistency, with a Cronbach’s alpha coefficient of 0.81 in this study.

### Extraversion personality inventory

This questionnaire, developed by Wang et al. (2011) based on the Chinese Big Five Personality Inventory (CBF-PI), was chosen for its specific focus on assessing extraversion traits, which are central to this study’s examination of personality as a mediator of altruistic behavior. It comprises a total of 40 questions, with 8 questions specifically designed to assess extraversion personality traits. Participants rated their responses on a 6-point scale ranging from “extremely non-compliant” to “extremely compliant.” The reliability coefficient for this inventory was calculated to be 0.82, indicating a high level of internal consistency.

### Perceived Social Support Scale

The Perceived Social Support Scale, originally developed by Zimet et al., and adapted for use in China by Huang et al. (1996), was selected for its relevance in assessing the role of perceived social support in promoting prosocial behaviors. This scale assesses perceived social support across three dimensions: family support, friend support, and other support, comprising a total of 12 questions. Respondents rate their level of agreement on a 7-point scale, ranging from “strongly disagree” to “strongly agree.” Higher scores indicate a greater perception of social support, which are hypothesized to be positively related to altruistic behavior. In this study, the scale demonstrated strong internal consistency, with a Cronbach’s alpha coefficient of 0.87.

### Altruistic Behavior Questionnaire

Developed by Li Yanfang in 2008, the Altruistic Behavior Questionnaire was selected for its comprehensive assessment of altruistic tendencies. The 22-item scale is organized into five dimensions: altruistic behavior responsibility, respect and care for others, care and concern for oneself, altruistic behavior performance, and selfish behavior and concepts. Items related to self-care and selfish behavior are reverse-scored to ensure accurate reflection of altruistic tendencies. Respondents rate their agreement on a 7-point scale, ranging from “very non-compliant” to “very compliant.” A higher total score indicates a greater level of altruistic behavior. The reliability of this scale, assessed using Cronbach’s alpha coefficient, was found to be 0.82 in the present study, suggesting strong internal consistency.

### Data analysis and common method bias testing

SPSS 26.0 software was employed to conduct a series of statistical analyses, including descriptive statistics, correlation analysis, and regression analysis. To assess mediation effects, the Process V4.0 plugin was utilized for mediation model analysis.

Given that data were collected via self-report, it was necessary to perform a common method bias test to ensure the validity of the findings. The Harman single-factor test was used to conduct an exploratory factor analysis (EFA). Specifically, the unrotated factor solution was examined to determine whether a single factor accounted for the majority of the variance. The results identified 11 factors with eigenvalues greater than 1. Importantly, the first factor explained only 20.17% of the total variance, which is well below the critical threshold of 40%. This finding suggests the absence of significant common method bias in this study (Zhou and Long, 2004).

## Results

### Descriptive statistics and related analysis

Descriptive statistics and Pearson’s correlations for the main variables are presented in Table 1. The results indicate that Family function is positively correlated with extraversion personality (*p* < 0.01), Perceived Social Support (*p* < 0.001), and Altruistic Behavior (*p* < 0.001). Moreover, extraversion personality is positively correlated with Perceived Social Support and Altruistic Behavior (*p* < 0.001) respectively. Additionally, Perceived Social Support is significantly positively correlated with Altruistic Behavior (*p* < 0.001).

**Table 1:** Descriptive statistics and correlation coefficient matrix (*N* = 972).

	*M* ± SD	1	2	3	4
1. Family function	5.31 ± 2.63	1			
2. Extraversion personality	29.85 ± 7.27	0.10^**^	1		
3. Perceived social support	59.04 ± 11.02	0.52^***^	0.29^***^	1	
4. Altruistic behavior	102.87 ± 8.66	0.14^***^	0.24^***^	0.32^***^	1

**p < 0.01, ***p < 0.001.

### Regression and mediation effect analysis

Building on the results of the correlation analysis and considering the statistical assumptions required for mediation analysis, further mediation analysis involving extraversion personality and Perceived Social Support can be explored (Wen and Ye, 2014). In this model, Family function serves as the independent variable, Altruistic Behavior as the dependent variable, and extraversion personality and Perceived Social Support as the mediating variables.

The mediation effects were analyzed using the bias-corrected percentile Bootstrap method within the SPSS macro program Process developed by Hayes. Specifically, Model 6, designed for examining chain mediation effects, was employed for testing, with 5,000 bootstrap samples and a 95% confidence interval.

The regression analysis revealed significant relationships between the variables (see Table 2). The findings indicate that Family function significantly predicts extraversion personality (*β* = 0.10, *p* < 0.01), and Perceived Social Support (*β* = 0.49, *p* < 0.001). Moreover, Perceived Social Support positively predicts Altruistic Behavior (*β* = 0.28, *p* < 0.001), while extraversion personality is positively associated with Perceived Social Support (*β* = 0.24, *p* < 0.001). Additionally, extraversion personality demonstrates a positive predictive effect on Altruistic Behavior (*β* = 0.16, *p* < 0.001).

**Table 2:** Model for regression analysis between variables (*N* = 972).

Predictor variable	Outcome variable: EP		Outcome variable: PSS		Outcome variable: AB	
	*β* (Boot SE)	95%BootCI	*β* (Boot SE)	95%BootCI	*β* (Boot SE)	95%BootCI
FF	0.10 (0.10)	[0.08,0.46]	0.49 (0.12)	[1.82,2.29]	0.02 (0.13)	[−0.32,0.17]
EP			0.24 (0.04)	[0.28,0.46]	0.16 (0.04)	[0.11,0.27]
PSS					0.28 (0.03)	[0.16,0.28]
	*R*^2^ = 0.01		*R*^2^ = 0.33		*R*^2^ = 0.12	
	*F* = 7.81**		*F* = 197.75***		*F* = 38.12***	

*p < 0.01, ***p < 0.001. FF, Family Function; EP, Extraversion Personality; PSS, Perceived Social Support; AB, Altruistic Behavior.

**Figure 2:**
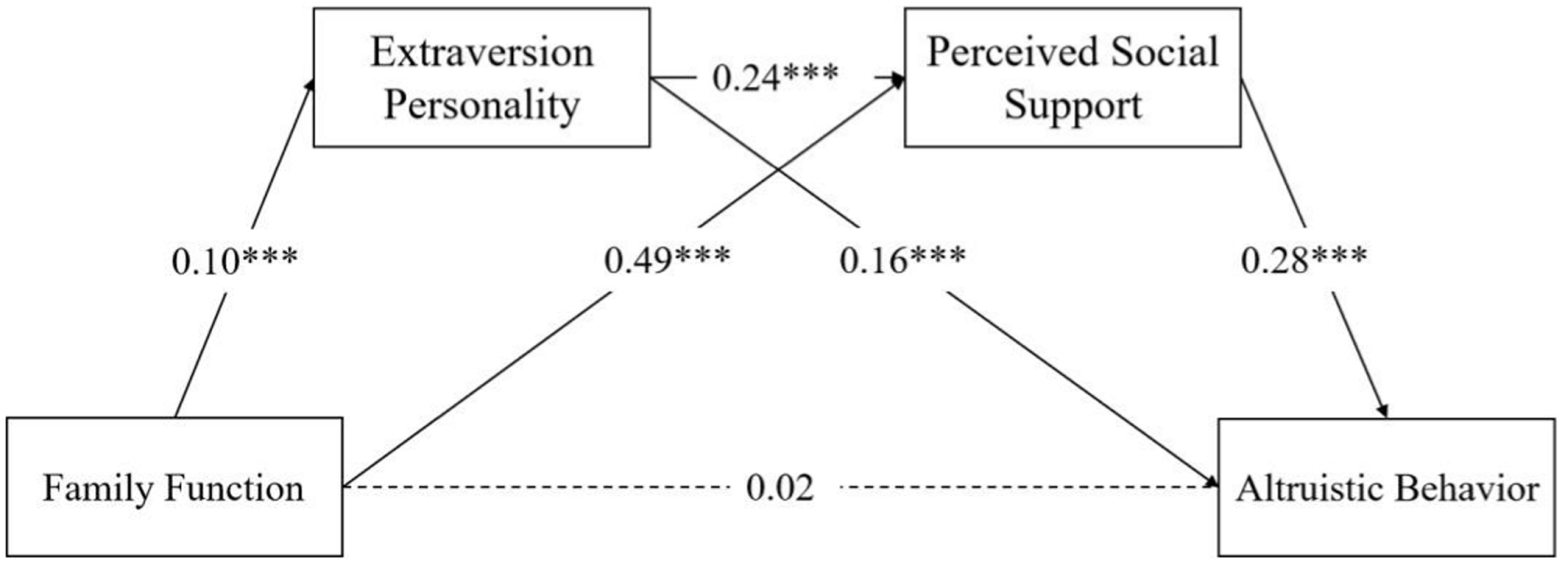
Mediating effects of extraversion personality and perceived social support.

The mediation analysis provided further insights into the relationships between family function, extraversion personality, perceived social support, and altruistic behavior. While the direct effect of family function on altruistic behavior is not significant. However, family function indirectly influences altruistic behavior through extraversion personality and Perceived Social Support. Specifically, extraversion personality acts as a mediator between family function and altruistic behavior, with a mediating effect of 0.16, explaining 48.48% of the total effect. Similarly, perceived social support also serves as a mediator between family function and altruistic behavior, with a mediating effect of 0.14, explaining 42.42% of the total effect. Additionally, a chain-mediated effect of extraversion personality and perceived social support on altruistic behavior was observed, with a mediating effect of 0.01, accounting for 3.03% of the total effect. These findings highlight the complex, indirect pathways through which family function influences altruistic behavior (see Table 3; Figure 2).

**Table 3:** Mediation effect analysis (*N* = 972).

Model pathways	Effect size	Boot SE	Boot LLCI	Boot ULCI
FF → AB	0.02	0.13	−0.32	0.17
FF → EP → AB	0.16	0.01	0.01	0.03
FF → PSS → AB	0.14	0.02	0.10	0.18
FF → EP → PSS → AB	0.01	0.01	0.01	0.01

FF, Family Function; EP, Extraversion Personality; PSS, Perceived Social Support; AB, Altruistic Behavior.

## Discussion

The findings of this study indicate a significant positive correlation between family function and altruistic behavior, aligning with prior research outcomes (Cui et al., 2022; Mei, 2023). However, upon incorporating the mediating variables of extraversion personality and perceived social support, the direct predictive effect of family function on altruistic behavior became nonsignificant. This may suggest that the influence of family function on altruistic behavior involves a multifaceted process.

A positive outlook on the external world may incline individuals toward understanding and assisting others in interpersonal interactions. According to social learning theory, individuals assimilate interaction patterns observed within their families, internalizing them as models for their own social conduct (Markiewicz et al., 2001). Robust Family function can imbue individuals with valuable attributes, such as adept problem-solving and communication skills, facilitating constructive interpersonal engagement among teenagers (Tan and Zhou, 2008). This enhanced interpersonal trust fosters the emergence of altruistic behavior (Guo et al., 2018).

The research findings suggest that the positive predictive effect of family function on altruistic behavior can occur through direct pathways or indirectly by enhancing adolescents’ perception of social support. In this study, the indirect effects of family function on altruistic behavior through extraversion personality and perceived social support were tested using a chain mediation model. The results suggest that family function influences altruistic behavior not only directly but also through these two mediating variables. Specifically, extraversion personality serves as the first mediator, shaping adolescents’ social engagement and leading them to perceive higher levels of social support, which subsequently increases their likelihood of engaging in altruistic behavior. This sequential, or chain, mediation highlights the process by which family environments contribute to the development of individual personality traits that, in turn, foster the social resources necessary for altruism.

The indirect effect through extraversion alone suggests that family function promotes prosocial tendencies by cultivating traits such as sociability and emotional expressiveness. Adolescents from supportive family backgrounds are more likely to develop extraverted characteristics, which make them more prone to altruism in social settings. Furthermore, the indirect effect of perceived social support on altruistic behavior underscores the importance of social networks. Extraverted individuals, because of their active social lives, tend to perceive greater social support, which motivates them to reciprocate through prosocial acts such as helping and comforting others. This aligns with social norm theory, which posits that individuals tend to maintain a balance between giving and receiving within social relationships (Cialdini and Trost, 1998).

Numerous studies have demonstrated that individuals receiving support and understanding from society can augment their internal psychological resources, fostering positive behaviors like altruism, mitigating negative emotional experiences, and fostering the holistic development of individuals (Gest et al., 2001; Xu et al., 2022). A functional family not only plays a pivotal role in the positive and healthy personality development of its members (Khodarahimi, 2011) but also furnishes them with a robust and stable sense of security, enabling them to perceive altruistic support from the family while cultivating optimism and confidence. Moreover, they can actively and confidently engage with classmates and teachers at school, thereby experiencing increased social support from peers and educators. When adolescents perceive heightened levels of social support, they can cultivate a profound sense of belonging (Wu, 2022), interpret their environment positively, exhibit empathy, attend to the needs of others, and, in line with social norms theory, develop a moral commitment and sense of obligation toward those providing social support, thus fostering altruistic behavior.

Regarding the influence of family factors on altruistic behavior, some researchers argue that it operates through personal traits (Zhang and Li, 2020). However, the impact of personal traits on altruistic behavior is still in its nascent stages. This study reveals that the positive predictive effect of family function on altruistic behavior can be mediated by the extraverted personality of adolescents, with extraversion personality playing a mediating role between the two. These findings contribute to a deeper understanding of this field.

The influence of family on personality is significant (Cang, 2023). Research indicates that personality partially mediates the relationship between family function and internalizing problematic behavior, and completely mediates the relationship between intimacy and externalizing problematic behavior (Li et al., 2008). However, internalizing and externalizing problems are not conducive to altruistic behavior, and personality factors are crucial influencing factors of altruistic behavior. A closer family relationship correlates with heightened interest and participation in the outside world, fewer family conflicts, more outgoing personality traits, and better interpersonal adaptability. The family environment indirectly shapes interpersonal adaptability through personality (Ma et al., 2014), thereby impacting altruistic behavior.

Additionally, this study finds that Family function could influence Altruistic Behavior through a chain mediated effect of extraversion personality and Perceived Social Support. As a crucial environment in an individual’s growth and development, the family is also the primary venue for personality formation and development (Kimayer and Young, 1998). Numerous studies have concluded that a harmonious, joyful, comfortable, and loving family environment positively impacts the formation and development of individual personality (Xie et al., 2008; He and Liu, 2008; Wei and Chen, 2010) posit that family function directly affects an individual’s personality development. Extraversion personality traits in personality significantly predict the score of network altruistic behavior (Liu, 2019).

Furthermore, personality traits influence the self-efficacy of high school students, often remaining relatively stable and having a strong predictive effect on self-efficacy (Wang, 2002; Li and Kuang, 2010). Individuals with high self-efficacy are more inclined to engage in altruistic behavior (Liu et al., 2021). According to the Mc Crae and Costa personality Big Five structure model, individuals with extroverted personalities are more likely to perceive social support around them, provide positive feedback, and engage in altruistic behavior.

While this study provides valuable insights into the relationships between family function, extraversion personality, perceived social support, and altruistic behavior in adolescents, several limitations should be acknowledged. First, the cross-sectional design of the study limits our ability to draw causal inferences between the variables. Although mediation analysis allowed for exploring potential pathways, future research should employ longitudinal designs to better establish the causal directions of these relationships and understand how they evolve over time. Second, the study relies on self-report measures, which carry the risk of response biases, including social desirability and subjective interpretation. Despite our efforts to mitigate this limitation through the use of validated instruments and testing for common method bias, these issues remain inherent in self-reported data. Future studies could complement self-report data with more objective measures, such as behavioral observations or peer assessments, to validate and strengthen the findings. Third, the study’s findings may be limited in generalizability due to the specific cultural context of the sample, which was drawn from high school students in Guangdong Province, China. While this regional focus offers unique insights, the cultural and socioeconomic differences may limit the applicability of the results to other populations. We encourage future research to replicate this study in different cultural and geographic contexts to examine the universality of these relationships. Forth, we acknowledge the possibility of unmeasured variables that could have influenced the findings. Factors such as socioeconomic status, parental education, peer influence, and other psychological constructs like empathy or emotional regulation were not included in this study. Future research should consider incorporating these variables to provide a more comprehensive understanding of the factors contributing to altruistic behavior. Fifth, although gender may play a moderating role in the relationships examined, we did not analyze gender moderation in the current study. Future research should investigate whether gender differences influence the mediating roles of extraversion and perceived social support in the link between family function and altruistic behavior. Exploring gender as a moderating variable could provide a more nuanced understanding of the dynamics at play and offer targeted insights for interventions. Future studies could also expand on this work by exploring other potential mediators and moderators that may impact the relationship between family function and altruistic behavior. For example, examining the role of personality traits beyond extraversion or considering the influence of social environment variables could yield new insights.

### Implications for practice and policy

The findings of this study offer valuable insights for developing interventions and policies aimed at fostering altruism and promoting positive youth development.

First, family-focused interventions should be prioritized to improve family function, as supportive family environments significantly influence the development of altruistic behavior in adolescents. Parenting education programs, family therapy, and support services for families experiencing difficulties may help improve family dynamics and promote positive youth development.

Second, interventions aimed at promoting positive personality traits, such as extraversion personality, should be integrated into school-based programs and community initiatives. Social skills training, group activities, and mentoring programs may help adolescents develop interpersonal competencies and confidence in social interactions, encouraging prosocial behaviors. These programs may also support the development of leadership skills and empathy, reinforcing altruistic tendencies.

Third, efforts to strengthen social support networks among adolescents should be prioritized to provide them with the necessary resources and encouragement to engage in helping behaviors. School-based support services, community engagement programs, and peer support networks may help create a supportive environment for adolescents to develop and express their altruistic tendencies. For instance, volunteering opportunities and community service projects can be integrated into school curricula, promoting both social connection and altruism.

Fourth, policymakers should consider the role of Family function, personality traits, and social support networks in shaping altruistic behavior when designing policies and initiatives aimed at promoting positive youth development. By addressing both individual and environmental factors, policymakers can create a holistic approach to promoting altruism and fostering prosocial behavior among adolescents. For example, government-sponsored parenting workshops or community centers that facilitate youth engagement could significantly enhance the social and emotional development of adolescents.

Looking ahead, future research could explore the long-term impact of such interventions on altruistic behavior and how different cultural or socioeconomic contexts may affect the outcomes. Moreover, practical applications of these findings should extend to diverse settings, such as rural or underserved communities, to examine the scalability and effectiveness of these programs across various populations.

In conclusion, the findings of this study highlight the importance of considering both individual and environmental factors in understanding and promoting altruistic behavior among adolescents. By fostering supportive family environments, promoting positive personality traits, and strengthening social support networks, policymakers, educators, and practitioners can contribute to the promotion of altruism and the well-being of adolescents in society.

## Conclusion

This study provides valuable insights into the factors influencing altruistic behavior among adolescents. By examining the interplay between Family function, personality traits, perceived social support, and altruism, we have uncovered complex relationships that contribute to our understanding of prosocial behaviors in this population.

The findings suggest that while Family function does not directly predict altruistic behavior, it exerts indirect effects through extraversion personality and perceived social support. Extraverted individuals are more likely to engage in altruistic acts, highlighting the importance of individual disposition in shaping prosocial behaviors. Moreover, perceived social support, particularly from family and friends, plays a crucial role in fostering altruistic tendencies among adolescents.

The mediation analyses revealed intricate pathways through which Family function influences altruistic behavior, underscoring the need for a multidimensional approach to understanding prosocial behaviors. By considering both individual characteristics and social contexts, we gain a more comprehensive understanding of the mechanisms underlying altruism among adolescents.

These findings have important implications for interventions aimed at promoting altruistic behaviors and fostering supportive environments for adolescents. By addressing factors such as family dynamics, personality traits, and social support networks, we can create environments that nurture altruism and contribute to the well-being of adolescents and their communities.

## Data Availability

The raw data supporting the conclusions of this article will be made available by the authors, without undue reservation.
